# Enhancing Micropropagation of *Adenophora liliifolia*: Insights from PGRs, Natural Extracts, and pH Optimization

**DOI:** 10.3390/plants13131735

**Published:** 2024-06-23

**Authors:** Zsófia Kovács, Liz Kelly Portocarrero, Péter Honfi, Ildikó Kohut, Eman Abdelhakim Eisa, Andrea Tilly-Mándy

**Affiliations:** 1 HUN-REN Center for Agricultural Research, Institute for Soil Sciences, Department of Soil Biology, Herman Ottó út 15, H-1022 Budapest, Hungary; 2 Department of Floriculture and Dendrology, The Hungarian University of Agriculture and Life Science (MATE), 1118 Budapest, Hungaryhonfi.peter@uni-mate.hu (P.H.);; 3 Agricultural Research Center (ARC), Botanical Gardens Research Department, Horticulture Research Institute, Giza 12619, Egypt

**Keywords:** *Adenophora liliifolia*, micropropagation, plant growth regulators (PGR), MS medium, AgNO_3_, NAA (1-Naphthaleneacetic acid), coconut water, pH optimization

## Abstract

The endangered plant species *Adenophora liliifolia* faces threats to its survival in the wild, necessitating the development of effective micropropagation techniques for potential reintroduction efforts. This study demonstrates that *Adenophora liliifolia* effectively reproduces on MS synthetic medium with diverse plant growth regulators (PGR) and natural extracts, facilitating swift micropropagation for potential future reintroduction endeavors. It highlights the substantial impact of PGR composition and natural extracts on the growth and development of *A. liliifolia*. The ideal growth medium for *A. liliifolia* was determined to be ½ MS with specific treatments. Additionally, incorporating silver nitrate (AgNO_3_) at 5 mg L^−1^ into the medium led to enhanced root formation and shoot length, albeit excessive concentrations adversely affected root development. Varying concentrations of NAA significantly affected different plant growth parameters, with the 0.1 mg L^−1^ treatment yielding comparable plant height to the control. Moreover, 50 mL L^−1^ of coconut water bolstered root formation, while 200 mL L^−1^ increased shoot formation during in vitro propagation. However, elevated doses of coconut water (CW) impeded root development but stimulated shoot growth. Experiments measuring chlorophyll a + b and carotenoid content indicated higher concentrations in the control group than differing levels of applied coconut water. Optimizing pH levels from 6.8–7 to 7.8–8.0 notably enhanced plant height and root formation, with significant carotenoid accumulation observed at pH 6.8–7. Soil samples from *A. liliifolia*’s natural habitat exhibited a pH of 6.65. Ultimately, the refined in vitro propagation protocol effectively propagated *A. liliifolia*, representing a pioneering effort and setting the stage for future restoration initiatives and conservation endeavors.

## 1. Introduction

The flora of Central Europe is currently influenced by a myriad of factors, including geography, geology, topography, climate, and vegetation history, and has had a significant impact from the glacial cycles of the Quaternary era. While certain plant species that are no longer present in Central Europe can still be found in regions like Eastern Asia, such as “*Platycladus orientalis*” (L.) Franco [1], Transcaucasia “*Pterocarya pterocarpa* (Michx.)” Kunth ex Iljinsk [2], and the Balkan Peninsula “*Picea omorika* (Pančić)” Purk [3], remnants from glacial or postglacial periods such as *Ligularia sibirica* and *Pedicularis sudetica* [4,5] coexist with newly emerged local endemic species like *Galium sudeticum*, *G. cracoviense*, *Cochlearia polonica,* and *Sorbus sudetica*. The distribution and composition of European species have been significantly impacted by climate change during the Quaternary Period [6] and human activities since the Neolithic Period [7].

*Adenophora liliifolia* (L.) Ledeb. ex A.DC., commonly known as Lilyleaf, Ladybells, or the fragrant bellflower, is a ‘perennial’ herbaceous plant ‘native’ to Asia, specifically China, Korea, and Japan. Belonging to the Campanulaceae family, *A. liliifolia* thrives in heliophilous, thermophilous, and basiphilous conditions with ample moisture [8]. Known for its fragrant bell-shaped flowers in shades of blue, purple, and white, this perennial species (up to 40 years) is insect-pollinated and exhibits a life strategy that is resilient to competition and stress but has limited capabilities for dispersing across great distances.

In Hungary, *A. liliifolia* is referred to as “csengettyűvirág” (EPPO, 2020) and is typically found in altitudes ranging from 100 to 680 m. Its habitats include Riparian mixed gallery forests in locations characterized by specific bedrock compositions such as organic-rich sediment, lacustrine and paludal clay, silt, calcareous mud, and peat. The species’ endangered status in Hungary and other Central European countries highlights the need for conservation efforts.

The central distribution of *A. liliifolia* spans from southern Siberia to Mongolia, China, Turkey, and various European countries [8,9,10,11]. Populations across Europe are facing declines due to factors like inadequate forest management and habitat alterations. According to Bilz [12], *A. liliifolia* is classified as a least-concern species in Europe due to its protection under the “Directive on the Conservation of Natural Habitats and Wild Fauna and Flora” (92/43/EEC). The emergence of eutrophicated high forests [13,14] and the replacement of coppices with conifers as a result of alterations in landscape management during the preceding two centuries resulted in the extinction of this species. In contemporary lowlands, *A. liliifolia* is located in limited populations that occupy ecotones, remains of light oak forests, and neighboring meadows. Furthermore, it is found in riparian forests that receive ample sunlight [15,16] and on rocky outcrops in beech woodlands at higher altitudes [17,18]. Because of its ecological requirements, *A. liliifolia* serves as an exceptional model plant for investigating the impacts of human activities on populations of related species [19].

Micropropagation or in vitro propagation techniques have been utilized to conserve threatened plant species like *A. liliifolia*. These techniques encompass apical or axillary meristem-derived micropropagation, somatic embryogenesis, cell culture methodologies, and cryopreservation techniques [20,21]. Establishing protocols for in vitro propagation is vital in preserving endangered plant species like *A. liliifolia* for future reintroduction efforts.

This study represents the first attempt at using micropropagation techniques on *Adenophora liliifolia* for conservation purposes. Specifically, seeds were used as explants on Murashige and Skoog (MS) synthetic medium, incorporated with various concentrations of AgNO_3_, NAA, CW, and adjusted pH in separate experiments. It aims to demonstrate successful multiplication, rooting efficiently, and acclimatization of *A. liliifolia* on a synthetic medium enhanced by phytohormones and natural extracts. Additionally, it seeks to explore the effects of different growth regulators on micropropagated plants’ development, determine suitable growing mediums, and establish an efficient micropropagation technique to produce in vitro plantlets for prospective reintroduction applications.

## 2. Results

### 2.1. Experiments Utilizing Various Medium Components

#### 2.1.1. Silver Nitrate (AgNO_3_)

AgNO_3_ is used in plant tissue culture to enhance root formation and shoot development by mitigating the effects of ethylene, a plant hormone that can inhibit growth. 

**Figure 1:**
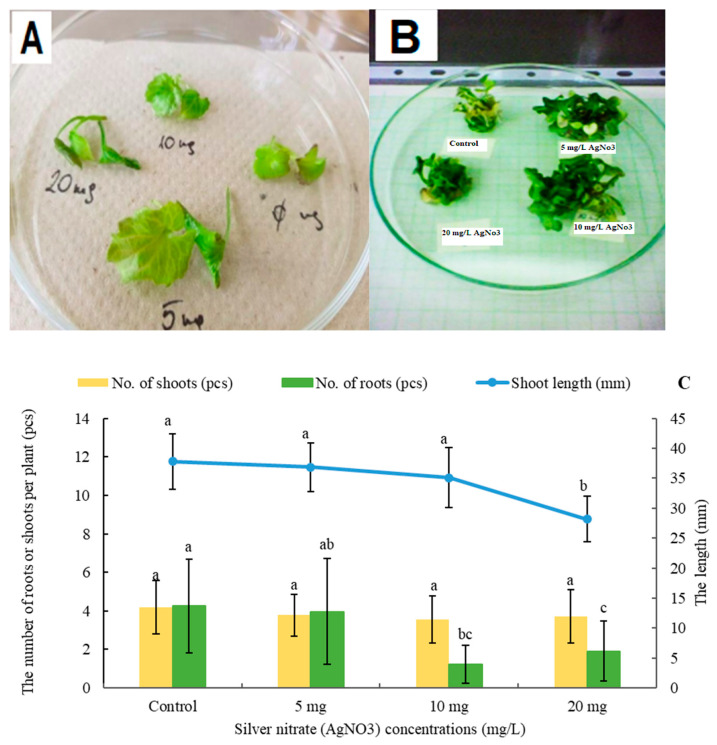
The effects of silver nitrate (AgNO_3_) application (control, 5, 10, and 20 mg L^−1^) in the in vitro medium on plant development (**A**,**B**); Shoot length [mm], No. of shoots, and No. of roots of *A. liliifolia* (**C**). Distinct letters represent statistically significant differences across groups (Games–Howell *p* < 0.05).

In this investigation, various concentrations of AgNO_3_ (5 mg L^−1^, 10 mg L^−1^, and 20 mg L^−1^) were tested within an in vitro medium to determine their impact on the morphological attributes of *A. liliifolia*. The parameters scrutinized included shoot length (mm), the number of shoots, and the number of roots. The results exhibited the most affected parameter was shoot length, exhibiting a gradual reduction from 37.8 mm (control group) to 28.2 mm at 20 mg L^−1^ of AgNO_3_; however, the number of shoots remained relatively stable across the treatments. Interestingly, the number of roots exhibited a significant decline with higher AgNO_3_ concentrations, from 4.3 (control) to 1.9 at 20 mg L^−1^, suggesting a relatively more significant impact on root development (Figure 1C).

Moreover, notable morphological variations were observed. In the control medium, plants exhibited thin, vitrified leaves. In contrast, plants in the 20 mg L^−1^ medium displayed reduced size, reflective leaves, and significant vitrification. On the other hand, the 5 mg L^−1^ and 10 mg L^−1^ treatments resulted in well-developed shoots with minimal vitrification. Statistical analysis indicated that the control treatment was comparable to the 5 mg L^−1^ and 10 mg L^−1^ treatments in terms of shoot length (Figure 1A,B). These multifaceted findings contribute to a comprehensive understanding of the nuanced effects of AgNO_3_ concentrations on the morphological features of *A. liliifolia* within an in vitro context.

#### 2.1.2. 1-Naphthaleneacetic Acid (NAA)

**Figure 2:**
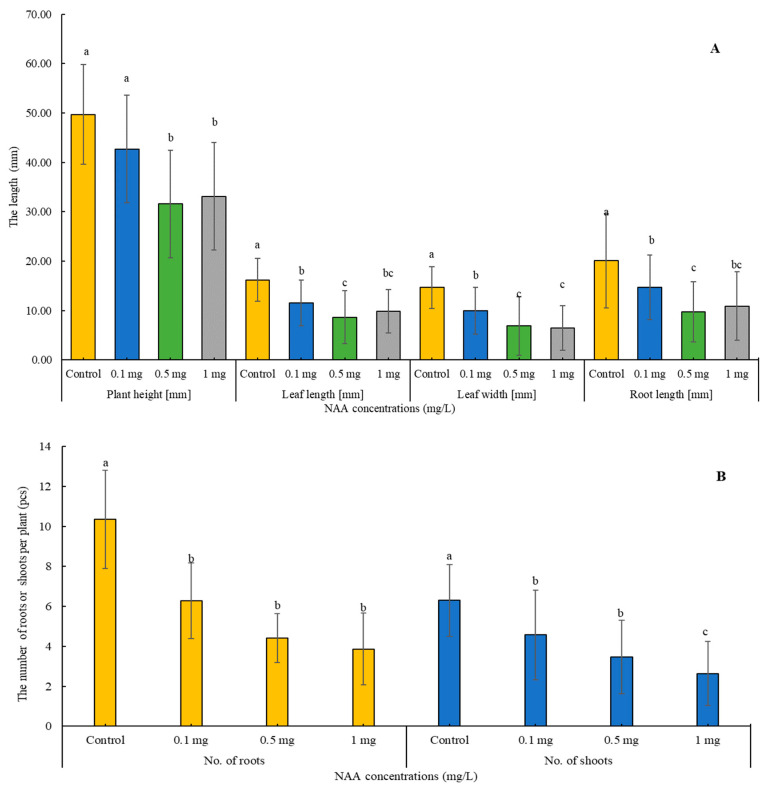
(**A**,**B**): Effect of 1-Naphthaleneacetic acid (NAA) application (control, 0.1, 0.5, and 1 mg L^−1^) in the in vitro medium on plant height (mm), leaf length (mm), leaf width (mm), root length (mm) (**A**), number of roots, and number of shoots of *A. liliifolia* (**B**). Distinct letters represent statistically significant differences across groups (Games–Howell, *p* < 0.05).

**Figure 3:**
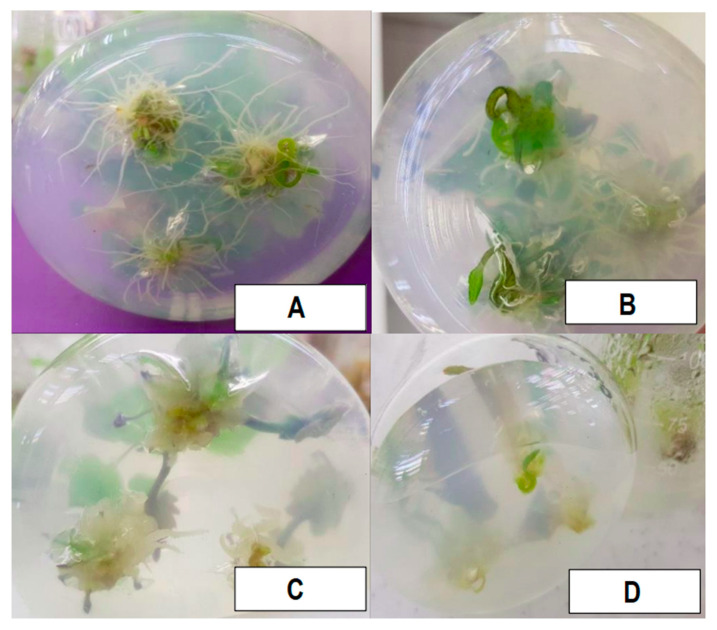
(**A**–**D**) Root formation in response to NAA concentrations of control, 0.1 mg L^−1^, 0.5 mg L^−1^, and 1 mg L^−1^.

The administration of 1-Naphthaleneacetic acid (NAA) at different concentrations (control, 0.1, 0.5, and 1 mg L^−1^) on the growing medium was ½ MS and induced notable alterations in the studied plant parameters with significant effects (*p* < 0.001) (Figure 2A,B). With increasing NAA concentration, a clear decline was observed in the quantities of roots, shoots, plant height, leaf length and width, and root length. The control group demonstrated superior values across all metrics, highlighting the significant influence of NAA treatments on plant growth and development. Conversely, the group treated with 1 mg/L of NAA displayed significant reductions in the number of roots and shoots by approximately 62.6% and 58.1%, respectively. Plant height, leaf length and width, and root length also experienced significant decreases of approximately 33.3%, 39.1%, 55.7%, and 45.7%, respectively. These findings underscore a dose–responsive relationship, indicating consistent decrements in growth parameters with increasing NAA concentration (Figure 3).

#### 2.1.3. Coconut Water (CW)

##### Coconut Water’s Impact on the Development of Plant Growth

**Figure 4:**
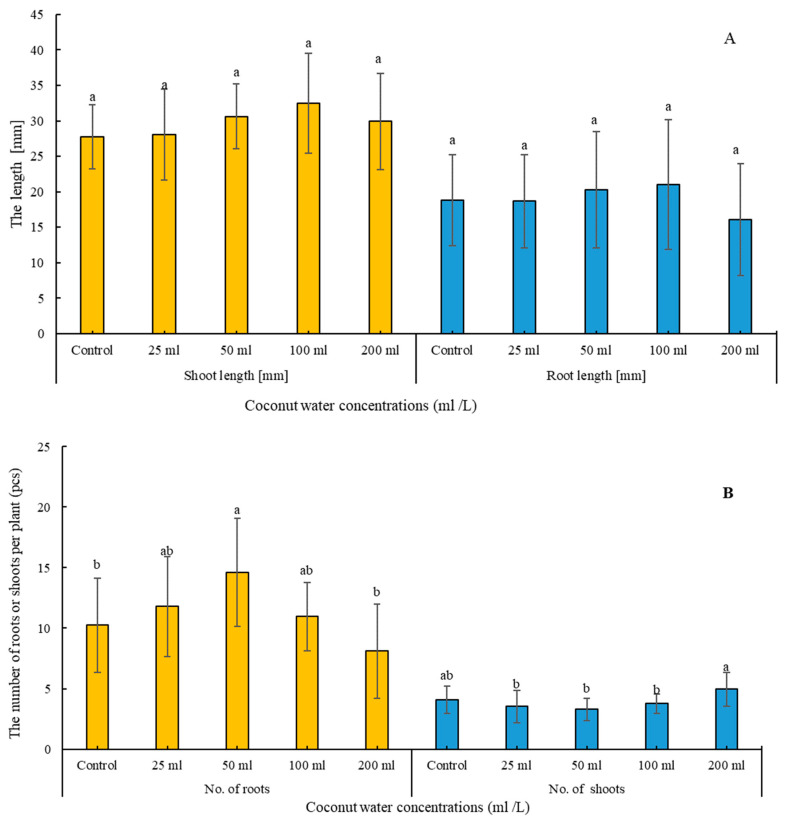
(**A**,**B**): Effect of coconut water concentrations (control, 25, 50, 100, 200 mL L^−1^) on shoot length [mm], root length [mm] (**A**), number of roots, and number of shoots of *Adenophora liliifolia* (**B**). Within a chart, values that are followed by the same letter are not statistically distinct from each other at a significance level of 5%, as determined by the Games–Howell’s post hoc test.

**Figure 5:**
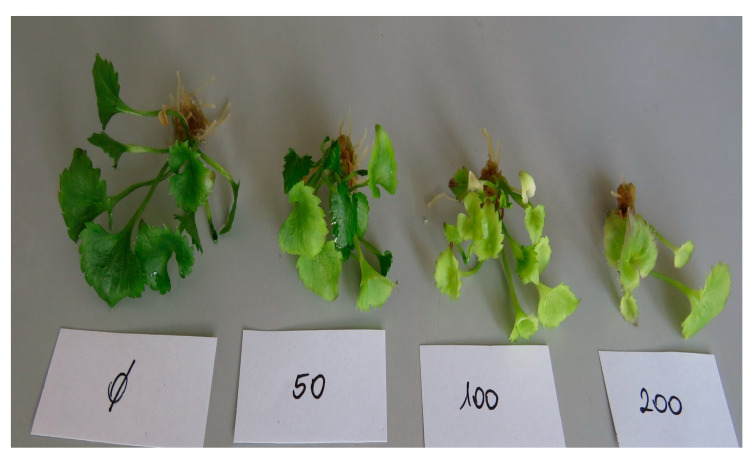
Effect of different coconut water levels (control, 25, 50, 100, 200 mL L^−1^) on plant characteristics of *A. liliifolia*.

The investigation into the effects of coconut water concentrations (control, 25, 50, 100, 200 mL L^−1^) on the growth parameters of *A. liliifolia* has yielded noteworthy outcomes, as demonstrated by the nuanced responses observed in the number of roots, number of shoots, plant height, and root length (Figure 4A,B) and (Figure 5). The control group exhibited a baseline performance, with 10.2 roots per plant, 4.1 shoots per plant, a plant height of 27.8 mm, and a root length of 18.9 mm. Intriguingly, the application of coconut water at 25 mL/L resulted in an increase in the number of roots (15.4% higher than the control) while maintaining a comparable number of shoots and plant height. The 50 mL/L concentration exhibited a substantial rise in both the number of roots (42.8% higher than the control) and plant height (10% higher than the control). However, a divergence was observed at 100 mL/L, where the number of shoots increased, while plant height and root length saw increments of 16.9% and 11.8%, respectively. The highest concentration of 200 mL/L, surprisingly, demonstrated a contrasting pattern, with a decrease in the number of roots (20.8% lower than the control) but a notable increase in the number of shoots (20.7% higher than the control). These findings suggest a concentration-dependent influence of coconut water on *A. liliifolia*, emphasizing the need for meticulous optimization to harness its potential benefits in promoting root and shoot development (Figure 5). 

##### Effect of Coconut Water on the Content of Chlorophyll and Carotenoids

**Figure 6:**
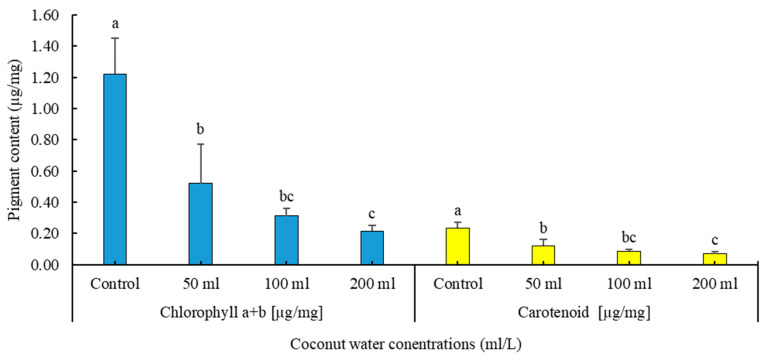
Effect of coconut water concentrations (control, 25, 50, 100, 200 mL/L) on chlorophyll a + b [µg/mg] and carotenoids [µg/mg] of *A. liliifolia*. Distinct letters represent statistically significant differences across groups (Tukey HSD test, *p* < 0.05).

The provided data in Figure 6 represent chlorophyll a + b and carotenoid content in *Adenophora liliifolia* following four different concentrations of coconut water. The coconut water treatments were set at 0 mL L^−1^ (control), 50 mL L^−1^, 100 mL L^−1^, and 200 mL L^−1^. The data show that both chlorophyll a + b and carotenoid levels exhibited variability among the different coconut water treatments. Specifically, the control treatment (without coconut water application) demonstrated the highest mean chlorophyll a + b content at 1.224 µg/mg. Conversely, as coconut water concentration increased, there was a notable decrease in both chlorophyll a + b and carotenoid levels. The lowest mean chlorophyll a + b content was recorded in the 200 mL L^−1^ treatment, registering at 0.215 µg/mg. Statistical analysis revealed significant differences in chlorophyll a + b content across the treatments. Similarly, the mean carotenoid content was highest in the control treatment, measuring 0.234 µg/mg, and lowest in the 200 mL L^−1^ treatment, at 0.070 µg/mg. Significant differences in carotenoid content among the treatments were also observed. These outcomes highlight the influence of coconut water concentrations on the photosynthetic pigment content of *Adenophora liliifolia*, suggesting a potential regulatory role of coconut water in plant physiological processes.

### 2.2. Optimizing pH Levels

#### 2.2.1. Developmental Impact of Varying pH Levels on Plants

**Figure 7:**
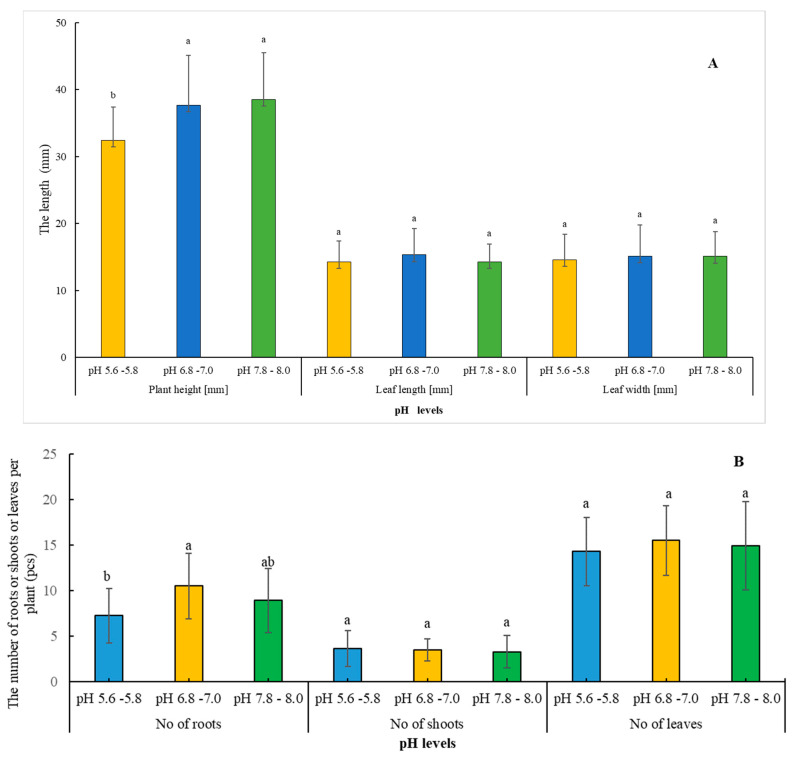
(**A**,**B**): Effect of three different pH levels (5.6–5.8, 6.8–7, and 7.8–8) on plant height [mm], leaf length [mm], leaf width [mm] (**A**), number of roots, number of shoots, and number of leaves of *A. liliifolia* (**B**). Distinct letters represent statistically significant differences across groups (Games–Howell, *p* < 0.05).

The provided data in Figure 7A,B represent the mean and standard deviation of several plant characteristics for *Adenophora liliifolia* following three different pH treatments. The pH treatments were set at 5.6–5.8, 6.8–7.0, and 7.8–8.0; the provided data show that the pH level significantly affects the growth and development of *Adenophora liliifolia*, with different pH treatments leading to varying plant characteristics. The highest mean values for plant height, leaf length, and width and the highest number of roots were observed in the pH 6.8–7.0 and 7.8–8.0 treatments, while the pH 5.6–5.8 treatment exhibited the lowest mean values. The variances in plant height and the quantity of roots among the treatments were found to be statistically significant. In contrast, leaf length and width, along with the counts of shoots and leaves, showed differences across the pH treatments, although these variances were not statistically significant.

#### 2.2.2. Pigment Content of *A. liliifolia* in Response to Variations in pH

**Figure 8:**
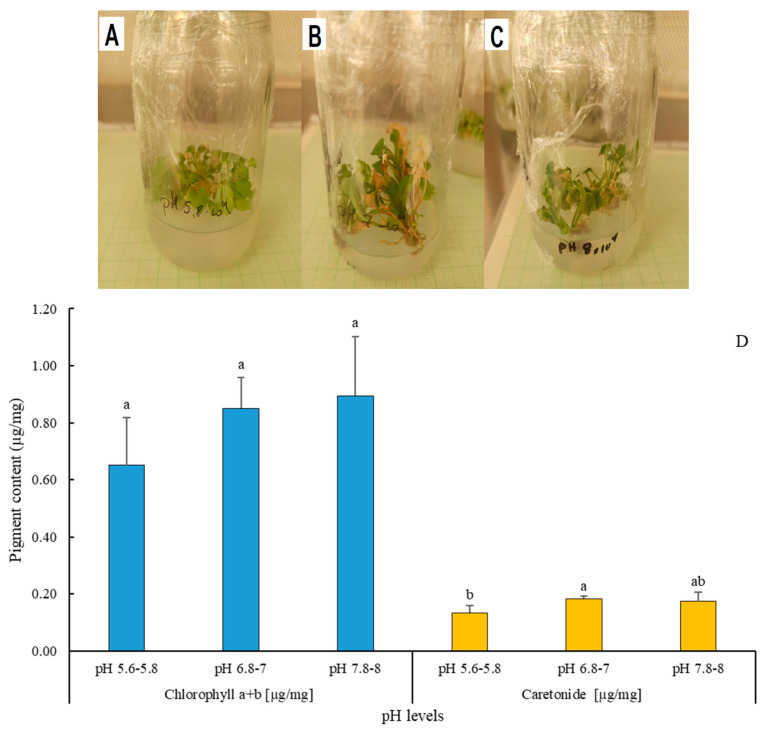
(**A**–**D**): Effect of three different pH levels (5.6–5.8, 6.8–7, and 7.8–8) on plant characteristics (**A**–**C**) and chlorophyll a + b [µg/mg] and carotenoids [µg/mg] of *A. liliifolia* (**D**). Distinct letters represent statistically significant differences across groups (Tukey HSD test, *p* < 0.05).

This study investigated the impact of varying pH levels on chlorophyll a + b and carotenoid content in *Adenophora liliifolia* (Figure 8D). The results revealed a noteworthy rise in carotenoid content at pH 6.8–7.0 compared to pH 7.8–8.0 (* *p* < 0.05), while chlorophyll a + b content increased consistently across all pH levels, though without significant differences. These findings underscore the plant’s sensitivity to pH fluctuations, offering insights into its physiological responses and ecological implications (Figure 8A–C).

## 3. Discussion

The effects of AgNO_3_ on growth parameters have been thoroughly investigated in the context of in vitro plant culture. Ethylene, a phytohormone, is essential for regulating various aspects of plant growth and development. Several studies have shown that the build-up of ethylene in culture media can lead to reduced growth and morphological changes in plants [22,23,24]. To counteract these effects, compounds such as AgNO_3_, a silver salt, have been utilized as anti-ethylene agents in vitro cultures [25,26,27]. Silver ions in AgNO_3_ can interfere with ethylene signaling by blocking ethylene receptors, thus inhibiting the plant’s response to ethylene [28,29,30,31]. Furthermore, silver nitrate can reduce ethylene production by inhibiting aminocyclopropane-1-carboxylic acid [32], a key component of the ethylene biosynthesis pathway, while promoting cell division and growth through the stimulation of polyamines [32,33,34]. Different concentrations of AgNO_3_ have varying effects on plant growth parameters. Our investigation into the optimal concentrations of AgNO_3_ revealed that a dose of up to 5 mg L^−1^ effectively prevents vitrification without compromising the number of roots and shoot length of plantlets. Contrarily, higher concentrations, such as 20 mg L^−1^, demonstrated varied effects on plant growth. Some studies reported increased longitudinal growth of hypocotyls with AgNO_3_ [29,35], while others found 20 mg/L effective in a nodal culture of *Vanilla planifolia* and *Vitex negundo* L. [36,37]. In our study, 20 mg/L maintained a number of shoots similar to the control but led to decreased shoot length and a reduced number of roots, consistent with findings by Tahoori et al. [35]. The variability in plant responses to AgNO_3_ appears to be species-specific and dependent on the concentration employed, suggesting the potential use of higher AgNO_3_ concentrations in culture media to preserve plantlets in vitro, thereby reducing the associated expenses.

“Naphthalene Acetic Acid” (NAA), a synthetic auxin, is a pivotal component influencing plant growth and development [38]. Research by Pant and Thapa [39] has shown that the effectiveness of shoot emergence in *Dendrobium primulinum Lindl* is dependent on the concentration and type of auxin present. Contrary to expectations, our findings regarding the impact of NAA addition to 1/2 MS medium did not exhibit a significant impact on the parameters studied. Notably, lower doses of NAA at 0.1 and 0.5 mg/L demonstrated greater efficacy in promoting shoot number, plant height, width, and leaf length compared to higher NAA concentrations, such as 1 mg/L, which seemed to impede the development of these characteristics, a trend similarly observed by Hartati et al. [40] in *Coelogyne pandurata* Lindley. Moreover, concerning root characteristics, supplementing NAA to 1/2 MS medium did not significantly influence root number or length compared to the control group. Interestingly, the minimal concentration of NAA at 0.1 mg/L yielded superior results in root development, aligning with a study by Ebrahimzadegan and Maroufi [41] on *Lallemantia iberica*, where the highest root growth was reported in a medium supplemented with 0.1 mg/L NAA. These results indicate that NAA application at specific concentrations can enhance root growth and differentiation, echoing the findings of previous studies [39,40,42,43,44].

Coconut water is widely utilized as a growth-enhancing constituent in tissue culture mediums [45]. Its incorporation into the media has been shown to facilitate organogenesis development, primarily attributed to the presence of gibberellins (GAs) within coconut water. Furthermore, coconut water exhibits growth-regulating properties akin to cytokinins [46], owing to its composition rich in amino acids, minerals, nucleic acids, purines, sugars, alcohols, vitamins, and phytohormones. Studies by Ichihashi and Islam [47] have demonstrated that the liquid endosperm of coconuts induces cell division and promotes morphogenesis in *Dendrobium ovatum* (L.) [48]. Based on our findings, media supplemented with higher concentrations of coconut water are recommended for enhancing shoot length and the proliferation of shoots, with the optimal levels being 100 mL for shoot length and 200 mL for accelerating the number of shoots (Figure 4). Additionally, auxins, cytokinins, and gibberellins, alongside other growth-promoting compounds present in coconut water, collaboratively influence various aspects of plant growth and development [49]. This comprehensive effect enhances several factors in tissue culture performance, including root length and biomass [50]. Notably, coconut water has been reported to exert significant effects on root initiation and development, with auxins, particularly indole-3-acetic acid (IAA), playing a crucial role in promoting root growth and increasing the quantity of root hairs [51]. Conversely, cytokinins act as negative regulators of root growth in plants by inhibiting root cell division [52], which may explain our results demonstrating that root biomass achieved optimal values at 50 mL of coconut water, while root length was most enhanced at 100 mL (Figure 4).

Additionally, the impact of coconut water (CW) application on ‘chlorophyll’ a + b and ‘carotenoid’ levels is illustrated in Figure (6). Our data demonstrate a significant influence of coconut water concentration on the ‘chlorophyll’ a + b and ‘carotenoid’ content in *Adenophora liliifolia*. The highest mean values for chlorophyll a + b and carotenoids were observed in the control treatment (without the application of CW), whereas the lowest mean values were recorded in the 200 mL L^−1^ treatment. Statistically significant differences in chlorophyll a + b and carotenoid content were evident among the treatments. This phenomenon may be attributed to the fresh extract nature of CW, which requires sufficient time for fermentation before its effects on increasing chlorophyll in plants can be realized. Furthermore, our findings suggest that cytokinins and gibberellins present in coconut water may not directly support chlorophyll formation, consistent with the findings of Mawarn et al. [53] regarding the effect of CW on binahong “*Anredera cordifolia* L.” pigments. It is plausible that cytokinins and gibberellins, prominent in CW, primarily play roles in cell elongation and enlargement, particularly in stems [54].

The ‘pH’ of the growth medium in plant tissue cultures plays a pivotal role in various facets of explant development and growth. Sensitivity or tolerance to fluctuations in medium pH differs across species, reflecting their specific requirements [55]. Moreover, medium pH modulates nutrient availability, either facilitating or inhibiting it [56]. Pasternak and Steinmacher [57] emphasized the multifaceted influence of medium pH in tissue culture systems, highlighting its impact on in vitro shoot multiplication, floral and secondary metabolite development, organogenesis, adventitious root production, and cell division. Our study indicates that pH variations within the prescribed range (6.8–7 to 7.8–8) can exert discernible effects on plant height and root development in *Adenophora liliifolia*, while parameters such as leaf dimensions and shoot count remain relatively unaffected. Typically, medium pH in plant tissue culture is adjusted to 5.8 to 6.0, albeit specific adjustments are made in accordance with the particular plant species and intended objectives [58]. In light of complementary soil sample data from the natural habitats of *Adenophora liliifolia* in Dabas and Ócsa, where pH levels range from 6.6 to 6.7, with corresponding electrical conductivity (CE) values, optimizing the medium to a pH of 6.8–7 appears to be the most suitable approach. Del Campo [59] and Khalil [60] observed that pH levels above 6 stimulate carotenogenesis in Muriellopsis species, and *Dunaliella bardawil* and *Chlorella ellipsoidea*, respectively, a phenomenon mirrored in our results. Specifically, the pigmentation of *A. liliifolia*, including carotenoids, increased with rising pH values, peaking at pH 6.8–7, while chlorophyll a + b levels displayed a gradual increase across different pH levels without significant variation. Carotenoids play crucial roles as photoprotectants and antioxidants, stabilizing chlorophyll levels [61]. Didyk et al. [62] proposed that the influence of medium pH on pigmentation may be indirect and mediated through growth and physiological changes rather than directly affecting carotenoid biosynthesis. Further investigation is warranted to elucidate the physiological mechanisms underlying these responses.

## 4. Materials and Methods

Principally, the investigation was conducted at the Hungarian University of Agriculture and Life Sciences in Budapest’s Laboratory of Micropropagation, which is affiliated with the “Department of Floriculture and Dendrology”.

The investigation focused on *Adenophora liliifolia* seeds obtained from two distinct sources:

- Seed samples were collected from a small, randomly chosen natural population in Ócsa, encompassing multiple species. The population consisted of approximately 10 individuals dispersed throughout the region, inhabiting a riparian mixed-gallery forest habitat.
- Jelitto^®^ Seeds: Additionally, the seeds of *Adenophora liliifolia*, specifically designated as Item No. AA112, was acquired from Jelitto^®^ (Schwarzenbach an der Saale, Germany). The seeds were utilized as explants to initiate the culture, and subsequently, the sterilized plantlets were employed for further experimentation. Notably, the plantlets cultivated in a controlled environment were selected as the primary specimens, resulting in a total of sixty individuals derived from seeds produced under controlled conditions.

### 4.1. Elements of Culture Media (Utilized in the Micropropagation of Adenophora liliifolia)

(a) The culture medium was composed of ½ MS, MS/2 macronutrients, MS micronutrients, and MS vitamins, with the inclusion of 100 mg m-Inositol, 25 mg Fe-EDTA, 20 g sugar, and 7 g agar, and had a pH of 5.8.
  The experimental setup included the following concentrations of silver nitrate (AgNO_3_): the control concentration of 0 mg L^−1^ was compared to concentrations of 5 mg L^−1^, 10 mg L^−1^, and 20 mg L^−1^.
(b) The growth medium utilized was 1-Naphthaleneacetic acid (NAA), which particularly consisted of ½ MS. The experiment employed several amounts of NAA: the control (0 mg L^−1^); 0.1 mg L^−1^; 0.5 mg L^−1^; and 1 mg L^−1^.
(c) Coconut Water (CW): The culture medium for coconut water (CW) was prepared by combining half-strength Murashige and Skoog (½ MS) medium with additional MS micro and MS vitamin supplements. In addition, it was fortified with 50 mg of Fe-EDTA, 20 g of sugar, and 6 g of agar. Different amounts of CW were utilized in the experiment.
(d) The control groups were exposed to different concentrations: 0, 25, 50, 100, and 200 mL L^−1^.
  The pH gradient was produced by utilizing a growing medium composed of ½ MS. The pH was modified by the addition of 1 N KOH and/or 1 N HCl. The experiment employed the following pH intervals: 5.6–5.8, 6.8–7.0, and 7.8–8.0.

### 4.2. Plantlet Manipulations and Environmental Conditions in the Growth Room

The experiment was carried out inside a laminar airflow box (BA-900, manufactured by “Debreceni Finommechanikai Vállalat”, Debrecen, Hungary). Plantlets are nurtured on illumination provided from above growth racks. The environmental factors affecting growth are as follows: The temperature is 22 °C with a margin of error of 2 degrees. The photoperiod consists of 16 h of light followed by 8 h of darkness. The luminance level during multiplication is 3000 l×.

### 4.3. Vegetative Measurement

Plant Height: The measurement of the longest shoot in a single cluster, expressed in millimeters (mm); Shoot Count: The total ‘number’ of live ‘shoots’ produced by the starting plantlet (pcs); ‘Leaf’ Count: The total ‘number’ of living leaves generated by the starting plantlet (pcs); ‘Root’ Count: The quantity of ‘roots’ generated by each plant (pcs); Root Length: The measurement of the longest ‘root’ for each unique plant, expressed in (mm); Leaf Length: The measurement of the distance from the point where the leaf attaches to the stalk to the opposite end of the leaf, expressed in (mm); and Leaf Width: The maximum distance between two points on the edge of the leaf that are perpendicular to the length of the leaf (measured in mm).

### 4.4. Pigment Content Measurement

This study collected fresh-weight samples of stems and leaves for two treatments: coconut water and pH gradients. The fresh weight was measured using the method set by [63]. The samples were homogenized using an acetone solution with a concentration of 80% (*v*/*v*) and quartz sand. The homogenized suspension was kept undisturbed to facilitate the separation of tissue remnants and quartz sand.

The light absorbance of the solution was evaluated using a ‘Genesys’ 10vis type ‘spectrophotometer’ at three specific wavelengths: A480 nm, A644 nm, and A663 nm. The content of chlorophyll and carotenoids was determined using a formula:

$$Chlorophylla+b\left(\mu g/mg\right)=\frac{(20.2\times A_{644}+8.02\times A_{663})\times X\;mL}{Weight}$$

$$\mathrm{Carotenoids}\left(\mu g/mg\right)=\frac{5.01\times {\;A}_{480}\times \;X\;\mathrm{mL}}{Weight}$$

X = 10 milliliters for ‘coconut water’ and 5 mL for pH ‘gradients. The methodology followed Arnon’s principles.

### 4.5. Statistical Analysis

This research employed a randomized design and utilized “Microsoft Excel” for “Microsoft 365” MSO (Version 2109), wherein the experiments were conducted. The data were analyzed utilizing either one-way or two-way MANOVA with version 27.0.1 of IBM SPSS Statistics software. To assess the normality of the residuals, the Shapiro–Wilk Test or Skewness [64] and Kurtosis were utilized [65,66]. The Lambda of Box–Cox transformation was implemented in the presence of multivariate outliers [67]. The method of Levene [68] examined the homogeneity of variances. At a 5% probability level, means were compared using the “Tukey HSD” or “Games–Howell” post hoc test [69], based on the acceptance or violation of the homogeneity of variances.

## 5. Conclusions

This study reveals that *Adenophora liliifolia* efficiently multiplies on MS synthetic medium, supplemented with various plant growth regulators (PGR) and natural extracts. This micropropagation system is intended for future reintroduction efforts. Notably, the composition of PGR and natural extracts significantly impacts *A. liliifolia* growth. The recommended addition to the medium is AgNO_3_ at 5 mg/L. While this treatment produced an abundance of roots and extended shoot length, it also reduced vitrification. NAA treatments at different concentrations had varying effects on plant growth parameters. Additionally, coconut water doses influenced root and shoot numbers. The pH optimization from 6.8–7 to 7.8–8.0 led to better plant height and root development. Soil samples from *A. liliifolia’s* habitat indicated a pH of 6.65. Overall, this study provides a foundation for restoration programs and conservation efforts.

## Data Availability

The data are contained within this article and are available upon request.
